# Comparative Analysis of the Clustering Quality in Self-Organizing Maps for Human Posture Classification

**DOI:** 10.3390/s23187925

**Published:** 2023-09-15

**Authors:** Lisiane Esther Ekemeyong Awong, Teresa Zielinska

**Affiliations:** Faculty of Power and Aeronautical Engineering, Division of Theory of Machines and Robots, Warsaw University of Technology, 00-665 Warszawa, Poland; teresa.zielinska@pw.edu.pl

**Keywords:** clustering quality, self-organizing maps (SOM), human posture classification

## Abstract

The objective of this article is to develop a methodology for selecting the appropriate number of clusters to group and identify human postures using neural networks with unsupervised self-organizing maps. Although unsupervised clustering algorithms have proven effective in recognizing human postures, many works are limited to testing which data are correctly or incorrectly recognized. They often neglect the task of selecting the appropriate number of groups (where the number of clusters corresponds to the number of output neurons, i.e., the number of postures) using clustering quality assessments. The use of quality scores to determine the number of clusters frees the expert to make subjective decisions about the number of postures, enabling the use of unsupervised learning. Due to high dimensionality and data variability, expert decisions (referred to as data labeling) can be difficult and time-consuming. In our case, there is no manual labeling step. We introduce a new clustering quality score: the discriminant score (DS). We describe the process of selecting the most suitable number of postures using human activity records captured by RGB-D cameras. Comparative studies on the usefulness of popular clustering quality scores—such as the silhouette coefficient, Dunn index, Calinski–Harabasz index, Davies–Bouldin index, and DS—for posture classification tasks are presented, along with graphical illustrations of the results produced by DS. The findings show that DS offers good quality in posture recognition, effectively following postural transitions and similarities.

## 1. Introduction

Recognizing human posture is crucial in a variety of application areas. Maintaining proper posture is important for injury prevention and good performance during sports and fitness [1]. In the field of ergonomics, the design and assessment of interventions aimed at reducing the risk of occupational injuries and enhancing productivity can be achieved based on posture information [2]. Posture recognition also has significant applications in surveillance and security sectors [3], such as in biometric authentication and access control systems. In robotics, human posture recognition is relevant for planning the actions of assistive robots [4]. Advancements in computer vision and machine learning technologies facilitate the automatic recognition of human posture utilizing images, depth data, acceleration, rotation, and orientation data of the human body [5,6]. Several machine learning methods have been utilized in human posture recognition, such as support vector machines (SVMs) [7,8,9], probabilistic models [10,11], decision trees [12], and K-nearest neighbor (KNN) [13]. However, traditional methods struggle with managing data complexity and extracting semantic information. Deep learning networks, unlike traditional machine learning algorithms, are capable of abstracting complex patterns in data by leveraging low-level feature information embedded within the data [14]. However, deep learning typically demands substantial data and computational resources for good performance.

While classic clustering algorithms have their limitations, they remain proficient in human posture recognition [15]. These algorithms categorize data points into clusters based on shared characteristics (a data point corresponds to a specific posture captured at a particular time frame). The K-means clustering algorithm is widely used for partitioning data based on mean values. Alternatively, self-organizing maps (SOMs) employ neural networks with adaptive weights for data clustering [16,17]. During training, SOMs modify their weights to reduce the distance between the vector of weights and the input data vector. Trained SOMs efficiently group similar data points together; hence, they are suitable for analyzing and categorizing diverse postures in human posture recognition tasks. Recognizing actions by a sequence of postures does not require distinguishing postures between successive recording frames. In classic interpretation, posture is associated with kinematic configuration (e.g., the elbow joint directed ‘inwards’, the elbow joint ‘outwards’, maintaining an acute or open angle in the knee joint, holding the trunk upright or inclined, etc.). This does not mean that the angular positions are fixed; they vary within a range that maintains a certain posture. This can be observed with humanoid robots imitating human movements, as described in [18,19,20,21]. Over-segmenting postures by choosing an excessive number of clusters complicates recognition. Moreover, activities executed by diverse individuals or under varying conditions might be characterized by minutely different postural data. Excessive clustering might lead to these being grouped separately, resulting in varying postural sequences for identical activities. This makes the method overly sensitive to contextual changes. On the other hand, having too few clusters could neglect essential postures that are crucial to specific activities. In clustering analysis, determining the appropriate number of clusters is an important factor that remains significantly challenging, often requiring repeated application of the clustering algorithm with modified parameters [22]. The number of clusters impacts the clustering performance [23,24,25]. Methods that select this number without assessing the clustering quality are inadequate. Moreover, the spatial contexts of human activities, whether indoors or outdoors, introduce additional complexities. Additionally, relying solely on a single distance metric (e.g., the Euclidean distance) for cluster quality assessments may not be the most effective approach [26]. Therefore, efficient methods for selecting the appropriate number of clusters are necessary. This article introduces a new clustering quality score and applies it to the task of classifying human postures. Other commonly known scores are introduced for the first time in posture classification, considering the state of the art. Describing these known scores alongside the new one allows for appropriate comparisons and analyses. It allows for illustrating the properties of the new score in relation to the characteristics of the known scores.

## 2. State of the Art

Human activity recognition plays a pivotal role in human–robot interaction (HRI) systems [3,27]. It involves discerning sequences of postures, which can be identified with varying degrees of precision. Yet, for effective recognition of human activities based on posture, it is crucial to pinpoint distinct key postures. Despite advancements, current recognition techniques have yet to reach optimal performance and often struggle with insufficient quality. When utilizing SOMs, it is important to ensure that the algorithm produces meaningful and coherent clusters.

Clustering quality evaluation also provides additional insight into the underlying structure of data, which is especially important [28] in real-world applications. Quality assessment techniques can be divided into internal and external clustering validity methods [29]. Internal methods e.g., the silhouette coefficient, Dunn index, Davies–Bouldin index, and Calinski–Harabasz index, evaluate clustering quality based on the data distribution and inter-cluster relationships. External methods compare the clustering results with ground-truth data, gauging to what extent the resulting clusters match the external labels, e.g., accuracy, precision, recall, and entropy [30]. Many recent works offer various insights into the process of determining the number of clusters in a dataset. Reference [31] investigated various combinations of stopping rules and clustering algorithms in an effort to determine the number of clusters in an artificially generated dataset. Their findings suggest that the number of clusters is significantly influenced by the clustering algorithm choice. On the other hand, the clustering algorithm selection is difficult without cluster validation methods. Alexander et al. [32] proposed a novel approach to determine the most reliable number of clusters for a couple of unsupervised machine learning algorithms. This approach utilized a parametric model featuring a rate–distortion curve. The key problem involved the introduction of the cost parameter, which characterizes the data dimensionality and homogeneity. While this parameter influences clustering results, it also adds to the method’s complexity and makes the process more reliant on the data structure. Ramazan et al. [33] applied 4 indices to estimate the best number of clusters for the K-means and consensus clustering algorithms, namely, silhouette (SH), Calinski-–Harabasz (CH), Davies–Bouldin (DB), and consensus (CI). In reference [34], the authors demonstrated that clustering stability testing can help to estimate the correct number of clusters. An extensive overview of these methods is provided in reference [35]. However, the effectiveness of these methods is highly dependent on the distribution of the data and can be a disadvantage when the data do not form well-separated clusters or represent clusters of unequal density.

In the study presented in [36], self-organizing maps (SOMs) were used to identify sources of groundwater salinity using hydrochemical data. The clustering quality of this SOM algorithm was assessed using the silhouette score. The silhouette score was also employed to establish the superiority of the clustering quality of a structural self-organizing map (S-SOM) used for synoptic weather typing [37]. The research highlights the versatility of the silhouette score in processing various types of data. However, for more robust and reliable decisions, it is evident that additional cluster validity indices (CVIs) are essential. This was applied in works such as [31], where the silhouette and Dunn indices were used to validate the SOM and K-means algorithms for classifying employees based on their disciplinary abilities. Other works applied different CVIs, e.g., Xiao et al. [38] proposed a hierarchical K-means algorithm that incorporates the Davies–Bouldin index as a metric, enabling the efficient identification of the number of clusters while minimizing computation time and costs. Caglar et al. [39] showed the effectiveness of the Calinski–Harabasz index as a robust cluster validation measure; it surpassed the performance of the Jaccard index and F-score in the clustering evaluation of cervical cells. Some relevant works on human posture recognition have also employed the aforementioned CVI for both supervised and unsupervised learning techniques [40,41,42,43,44,45]. The clustering validation used in the literature applies diverse assumptions about factors determining good clustering, e.g., compactness and separation, which may not align with the structures of the data or the specific problem at hand. It usually provides a single solution as the appropriate number of clusters, even if several solutions may be equally valid, depending on the context.

Overall, determining the most suitable number of clusters is an ongoing research topic. The proposed methods are mostly dependent on the nature of the datasets and underlying assumptions. As a result, these methods are more likely effective as guidance frameworks, rather than definitive solutions. A review of the literature suggests that prior studies have employed a limited number of clustering evaluation validity scores, with minimal emphasis on domain knowledge or context of the specific problem. Furthermore, only a handful of these studies have applied these scores to SOM-based NNs.

This work is organized as follows: In Section 3, we discuss the relevance of the research problem and highlight the challenges and motivation of performing clustering quality, Section 4 describes the data acquisition process and introduces the learning process of the SOM neural network, Section 5 introduces the applied clustering validity scores, and Section 6 details the results. This work describes the impacts of different factors, on classification performance, including the number of clusters (equivalent to the number of output neurons) and the type of distance metric. Section 7 concludes the work by discussing the findings and their relevance for human posture classification. It addresses potential limitations, proposes future research directions, and summarizes the key contributions of this work.

## 3. Problem Statement

Supervised learning requires human experts to label the training dataset, meaning they must designate to which group or cluster each data point belongs to. The main challenge in analyzing postures is the high dimensionality and variability of the data. In the context of the considered problem, traditional supervised classification would compel experts to laboriously define (label) the postures. In contrast, clustering techniques eliminate the need for human labeling, which not only demands expertise but is also time-consuming and demands significant attention [46]. Moreover, human decisions are subjective. Unfortunately, unsupervised clustering methods utilized for recognizing postures can suffer from insufficient evaluations of results; therefore, efficient and problem-oriented quality assessments are essential. This is challenging due to the subjectivity in posture extraction. Additionally, human expertise alone cannot identify key characteristics of the data. To address these limitations, a methodology for selecting a suitable number of clusters and a comparative analysis of the clustering performances are presented in this work. This eliminates the need for manual labeling while ensuring proper posture classification performance. Unlike prior studies that utilized a limited number of clustering quality scores, our results are derived using 5 distinct CVIs, e.g., the discriminant score, silhouette coefficient (or silhouette score, SC), Dunn index (DI), Davies–Bouldin index (DB), and Calinski–Harabasz index (CH). Additionally, the classic quantization error (QE) is taken into account.

The NN was trained to recognize sequences of postures that are typical for selected human activities. During the testing phase, data from both the chosen activity and another related activity (featuring similar postures) were employed. The primary objective was to determine the suitable number of postures that best described the activities and allowed capturing posture transitions effectively. The clustering quality score allows for the study of the relationship between postures, shedding light on both feasible and infeasible posture transitions.

## 4. Data Gathering and the Posture Recognition Method

Two ZED RGB-D vision sensors were used to collect human 3D-skeleton data, consisting of body point coordinates. Two activities considered in this work are part of our larger dataset, which consists of over 20 human activities recorded in 2 different laboratories, considering cultural aspects and different scenarios. The laboratories are located in a European country and Japan. The presented research focuses solely on the methodology, excluding data processing, training, and testing of the applied neural network. These problems were comprehensively covered in our previous publication [47,48], which also included an assessment of clustering results, a computational efficiency evaluation, a comparison to other classification methods, and a parameter selection process. Data used in the presented research were collected from 4 healthy participants who executed each activity 3 times, resulting in 12 records for an activity. Considering the so-called training activity (TA), 10 recordings were used for training and 2 for testing. For the non-training activity (UA), 2 recordings were used for testing. Data were recorded at a rate of 15 fps, with a resolution of 3840×1080. The training dataset was composed of 25,048 sample frames; 19 human body points were chosen for posture representation. The training activity (TA), from which 10 recordings were used for training the NN, consisted of picking up an object from the floor, walking, and placing the object on the table. The second activity used for testing consisted of walking, grabbing the cart, and pulling the cart. The activities were recorded in the sagittal plane (camera 1) and at a viewing angle of $50^{\circ }$ degrees (camera 2). Further details on the setup are presented in [47].

SOMs, also known as Kohonen Maps, are effective tools for analyzing complex unlabeled data. The SOM-NN learns by establishing a configuration of neurons, where each neuron symbolizes a cluster (group of data points). Each neuron has a weight vector of the same dimensionality as the input data. Firstly, the random weights (e.g., using uniform or Gaussian distribution) are assigned to the neurons. These weights are then updated during the learning process to match the input data by finding the ‘winning neuron’, i.e., the neuron with weights that are closest to the input data vector in terms of considered distance. This process is iterated over all input data vectors, leading to distinct clusters that represent postures with similar features.

## 5. Applied Scores

As already mentioned, when applying the SOMs, it is imperative to use scores that help in selecting the most suitable number of postures for a given problem [49]. In this research, the most popular scores, namely the silhouette coefficient, Dunn index, Calinski–Harabasz index, Davies–Bouldin index, and quantization error were used together with the novel discriminant score. The silhouette coefficient takes into account the pairwise intra-cluster and inter-cluster distances for cluster quality assessments. The Dunn index identifies groups of clusters that exhibit both compactness and low variance among their members. The Calinski–Harabasz index is characterized by the ratio of inter-cluster dispersion to intra-cluster dispersion for all clusters. The Davies–Bouldin index expresses the similarity between clusters. The discriminant score allows for evaluating the separability of classes. Table 1 introduces the basic notation used for the clustering score definition.

**The silhouette coefficient:**

The silhouette coefficient [50] is a good tool for evaluating the performance of clustering algorithms especially in high-dimensional datasets where direct visualization of results is limited. A graphical representation of the coefficient allows visualizing how well data points fit into their assigned clusters. Let us denote by ${\bar{a}}_{m}^{c_{,}}\left(k\right)$ the average distance between the data point $f_{k}^{c_{m}}$ and all other data points $f_{kk}^{c_{m}}$ assigned to the same cluster (kk≠k):

(1) $${\bar{a}}_{m}^{c_{m}}\left(k\right)=\frac{1}{K_{m}-1}\sum_{kk=1}^{K_{m}}d^{Me}\left(f_{k}^{c_{m}},f_{kk}^{c_{m}}\right),\;kk\neq k$$

The minimum of the average distances between data point $f_{k}^{c_{m}}$ and the data points assigned to each other cluster is denoted by ${\bar{b}}_{min}^{c_{m}}\left(k\right)$ and obtained as follows:

(2) $${\bar{b}}_{min}^{c_{m}}\left(k\right)=\min_{m1}(\frac{1}{K_{m1}}\sum_{k1=1}^{K_{m1}}d^{Me}\left(f_{k}^{c_{m}},f_{k1}^{c_{m1}}\right))$$

where m1=1,…,M and m1≠m. the silhouette coefficient for data point $f_{k}^{c_{m}}$ assigned to cluster $c_{m}$ is expressed by:

(3) $$SC^{c_{m}}\left(k\right)=\frac{{\bar{a}}_{m}^{c_{m}}\left(k\right)-{\bar{b}}_{min}^{c_{m}}\left(k\right)}{\max({\bar{a}}_{m}^{c_{m}}\left(k\right),{\bar{b}}_{min}^{c_{m}}\left(k\right))}$$

The silhouette coefficient ranges between −1 and 1. A score of 1 indicates good classification, meaning a data point is closer to members of its own cluster than to those in other clusters. Near 0 is still a good classification, although distances between a data point and members of its own cluster and a neighboring cluster are close, implying the potential of cluster overlapping. A negative score signifies miss-classification, with the data point being closer to the other clusters.

**The Dunn index:** The Dunn index [51] aims to maximize the distance between clusters while minimizing the distance within clusters. Let $d_{max}^{Me}\left(ff^{c_{m}}\right)$ denote the maximum distance between any 2 data points, *k* and kk, assigned to the same cluster:

(4) $$d_{max}\left(k,kk\right)=\max_{k\neq kk}(d_{c_{m}}^{Me}\left(f_{k}^{c_{m}},f_{kk}^{c_{m}}\right))$$

Another value considered in this criterion is the minimum distance between the cluster centroids:

(5) $$dc_{min}=\min_{m\neq m1}(d_{c_{m}}^{Me}\left({\mathrm{cc}}_{m},{\mathrm{cc}}_{m1}\right))$$

The Dunn index (DI) is expressed by

(6) $$\begin{array}{c} \begin{array}{c} DI=\frac{dc_{min}}{d_{max}\left(k,kk\right)} \end{array} \end{array}$$

The DI ranges from 0 to *∞*. A higher DI indicates better clustering results, with well-separated clusters and minimal overlap.

**Davies–Bouldin index:** This index shows how well-separated and distinct the clusters are, taking into account the ‘within-class’ similarity and the ‘between-class’ similarity. The index takes into account the average distance from each data point in cluster $c_{m}$ to its centroid:

(7) $$\bar{av}\left(m\right)=\frac{1}{K_{m}}\sum_{k=1}^{K_{m}}d^{Me}\left(f_{k}^{c_{m}},{\mathrm{cc}}_{m}\right)$$

This average is calculated for each *M* cluster separately. The next parameter is the distance between the centroids of clusters:

(8) $$d\left(m,m1\right)=d^{Me}\left({\mathrm{cc}}_{m},{\mathrm{cc}}_{m1}\right)$$

where m≠m1. For each pair of clusters (denoted as clusters *m* and m1), the sum of the average distances is divided by the distance between their centroids:

(9) $$dav\left(m,m1\right)=\frac{\bar{av}\left(m\right)+\bar{av}\left(m1\right)}{d(m,m1)}$$

For each cluster *m*, the maximum of dav(m,m1) (m≠m1) is obtained. Summing these maxima across all clusters yields the Davies–Bouldin index:

(10) $$\begin{array}{c} \begin{array}{c} dav_{max}\left(m\right)=\max_{m1}\left(dav(m,m1)\right)\\ DBI=\sum_{m=1}^{M}dav_{max}\left(m\right) \end{array} \end{array}$$

h ranges from 0 to positive infinity. A smaller DBI indicates better clustering performance. Values close to 0 represent well-separated and distinct clusters. A higher DBI indicates poorer clustering results, where clusters may be overlapping or poorly separated.

**Calinski–Harabasz index:** The Calinski–Harabasz index [52] shows how well-separated and dense the clusters are. The clustering quality is characterized by the ratio of inter-cluster dispersion to intra-cluster dispersion, using the Euclidean distance.

(11) $$CH=\frac{\left[\sum_{m=1}^{M}K_{m}{∥{\mathrm{cc}}_{m}-c∥}^{2}\right]/\left(M-1\right)}{\left[\sum_{m=1}^{M}\sum_{j=1}^{K_{m}}{∥f_{k}^{c_{m}}-{\mathrm{cc}}_{m}∥}^{2}\right]/\left(N-M\right)}$$

where *M* is the number of clusters, $K_{m}$ is the number of samples in cluster *m*, $c_{m}$ is the centroid of cluster *m*, *c* is the centroid of the entire dataset, $f_{j}$ is the individual data sample in cluster *m*, and *N* is the total number of samples in the dataset (N > M). A higher CH index indicates better clustering performance.

**Quantization error:** The quantization error [53] is another measure used to evaluate the performance of the NN. It applies the cosine distance between each data point and its best matching unit (BMU), which creates a measure of dissimilarity between the data point and the learned representation in the SOM. Minimizing the quantization error helps to improve the accuracy and quality of clustering. The quantization error of a data point *j* is expressed by:

(12) $$QE\left(j\right)=|\underset{m}{d_{min}^{Me}}\left(f_{j},w_{m}\right){|}^{2}$$

where m=1,…,M and Me represent the cosine metric.

Unlike the other scores, the quantization error illustrates how the clustering quality changes over the training process. The better the concentration of data around BMU, the lower the error.

**Discriminant score:** As proposed by us, the discriminant score focuses on intra-cluster similarity and inter-cluster dissimilarity, enabling the evaluation of the clustering algorithm’s performance in separating data points into distinct classes. The discriminant score provides value for each data point (each posture). It is the normalized inverse of the posture distance from the cluster centroid. This value is divided by the sum of the minimum distances between this data point and the centroids of all clusters. It indicates how similar a posture is to the posture representing the cluster centroids, taking into account the dispersion of clusters (this is included in the denominator). This score can be obtained using cosine and Euclidean distances, as follows:

(13) $$\begin{array}{c} \begin{array}{c} d^{Cos}\left(f_{j},{\mathrm{cc}}_{m}\right)=1-\frac{f_{j}\cdot {\mathrm{cc}}_{m}}{∥f_{j}∥∥{\mathrm{cc}}_{m}∥}\\ d^{Euc}\left(f_{j},{\mathrm{cc}}_{m}\right)=\left|\right|f_{j}-{\mathrm{cc}}_{m}\left|\right| \end{array} \end{array}$$

The term $d^{Me}\left(f_{j},{\mathrm{cc}}_{m}\right)$ indicates how close the data vector $f_{j}$ is to the centroid ${\mathrm{cc}}_{m}$ of each cluster. A smaller $d^{Me}\left(f_{j},{\mathrm{cc}}_{m}\right)$ signifies better similarity. When the data vector overlaps with the cluster’s centroid, then the distance is equal to 0. To express how ‘strong’ the classification result is and compare it with outcomes from other clusters, we take the inverse of the distance and divide it by the sum of distances obtained for all data vectors. This is expressed by

(14) $$\begin{array}{c} \begin{array}{c} if\;\;\;d^{Me}\left(f_{j},{\mathrm{cc}}_{m}\right)\neq 0\\ then\;\;a^{Me}\left(f_{j},{\mathrm{cc}}_{m}\right)=\frac{1}{|d^{Me}\left(f_{j},{\mathrm{cc}}_{m}\right)|}\;\;\\ and\;\;DS_{m}\left(k\right)=\frac{a^{Me}\left(f_{j},{\mathrm{cc}}_{m}\right)}{\sum_{m=1}^{M}\left|d_{min}^{Me}\left(f_{j},{\mathrm{cc}}_{m}\right)\right|}\\ if\;\;d^{Me}\left(f_{j},{\mathrm{cc}}_{m}\right)=0\;then\;\;DS_{m}\left(k\right)=1 \end{array} \end{array}$$

The score takes values from 0 to 1. A higher value of $DS_{m}\left(k\right)$ means higher confidence in the correctness of the classification result. Hence, perfect similarity is 1 and no similarity is 0. The $DS_{m}\left(k\right)$ measures obtained for all clusters allow us to discern how similar or distinct the postures are. Moreover, the $DS_{m}\left(k\right)$ drawings describe the postural transitions, which will be presented in more detail in the following section.

## 6. Evaluation of Clustering Quality

### 6.1. Approach

Two activities—training (TA) and unseen non-training (UA)—were used to determine the appropriate number of characteristic postures (the number of clusters). For the considered TA activity, 4 configurations comprising 3, 4, 5, and 6 output neurons (clusters) were indicated as possible class numbers by a human expert. However, the expert did not assign the postures to the clusters.

The discriminant score was applied to NNs trained using both cosine distance and Euclidean distance as criteria for updating the weights. The remaining applied scores (CVIs) were computed for NNs trained using only the cosine distance. This is because such NNs demonstrate greater flexibility with a variable number of outputs. In contrast, the NN trained using the Euclidean distance tended to consistently identify only 3 clusters for the various expected clusters; therefore, subsequent clustering quality evaluation using DS was not needed. When determining the DI, the cosine distance was used for NNs trained with the cosine distance, and the Euclidean distance was used for NNs trained with the Euclidean distance. For other criteria with optional distance selections, the cosine distance was used, according to the literature.

### 6.2. Results

**Figure 1:**
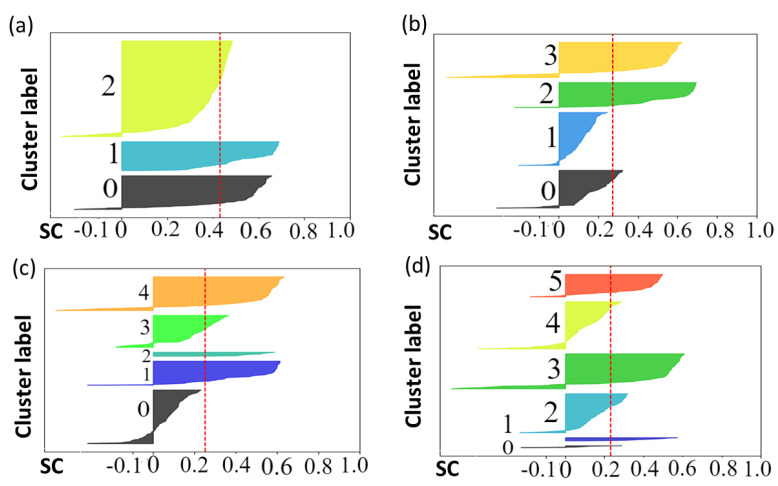
Silhouette score plots: (**a**) 3 clusters, (**b**) 4 clusters, (**c**) 5 clusters, and (**d**) 6 clusters.

**I. The silhouette coefficient (SC)**: Regarding the SC, each graph in Figure 1 represents the silhouette plots for 3, 4, 5, and 6 output configurations.

Each cluster is color-coded in the silhouette plot, with the red line indicating the average silhouette score. The plot with 3 clusters has the highest average score and minimal misclassification, particularly in cluster 1. Plots with 4 and 5 clusters show little misclassification and lower average SCs, while plots with 6 clusters reveal unbalanced data sizes. Despite all clusters exceeding the average score, clusters 0 and 1 are significantly smaller.

**Figure 2:**
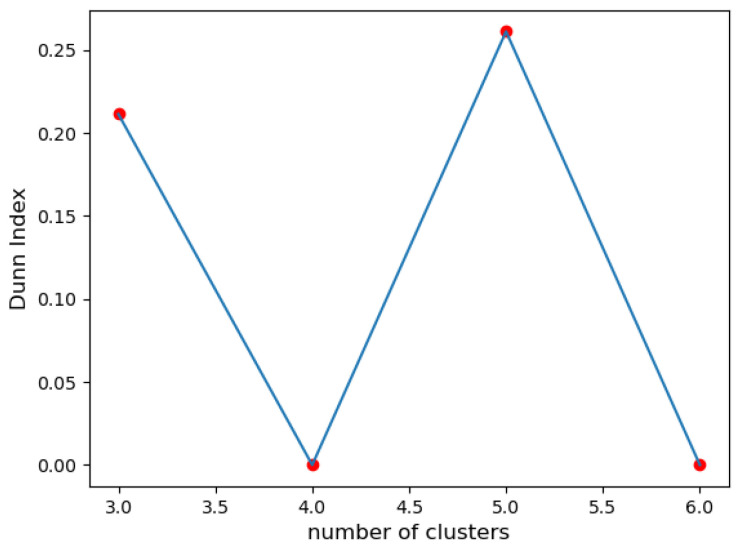
Dunn index for 3, 4, 5, and 6 clusters.

**II. The Dunn index:** The analysis shows that the configuration with 5 clusters has the highest DI (Figure 2), which means better clustering quality due to better separation and compactness. The configuration with 3 clusters also has a high index, indicating good cluster separation.

**Figure 3:**
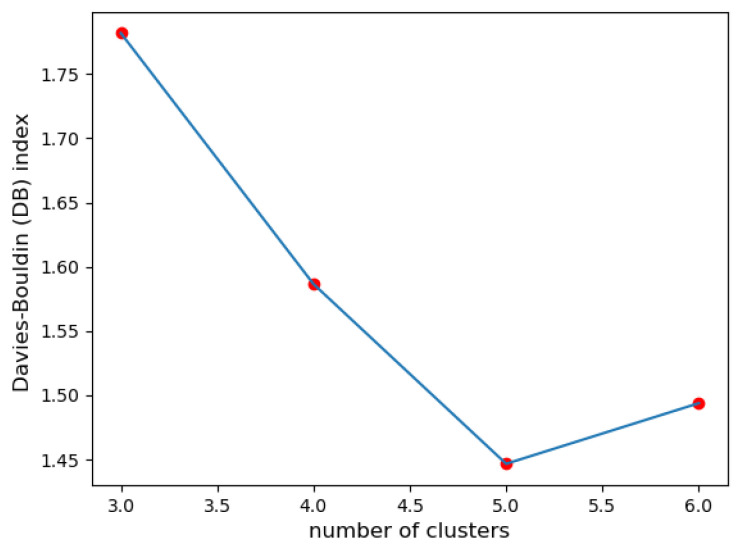
DB index for 3, 4, 5, and 6 clusters.

**III. Davies–Bouldin index:** Upon evaluating the quality of the clustering solutions with different cluster numbers (3, 4, 5, and 6), the index for the solution with 5 clusters was the lowest compared to the other configurations (see Figure 3). This suggests that clustering with 5 clusters leads to better separation and compactness of clusters.

**Figure 4:**
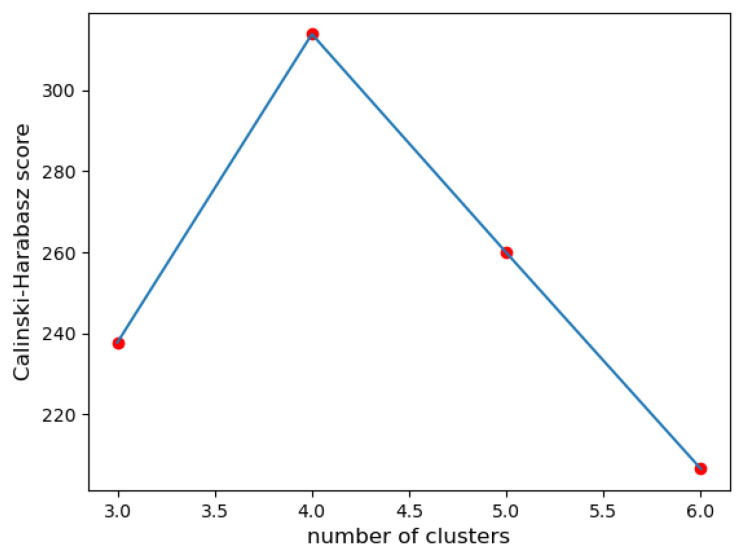
The Calinski-Harabasz index for 3, 4, 5, and 6 clusters.

**IV. Calinski–Harabasz index (CH)**: When evaluating the CH index, the configuration with 4 clusters is the best compared to the others. It has the highest value in comparison to the other configurations (see Figure 4). Certainly, a higher CH index value means that the clusters are dense and well-separated. However, there is no ‘acceptable’ cut-off value, and we need to choose the solution that provides a peak or at least an abrupt elbow on the line plot of CH indices.

**Figure 5:**
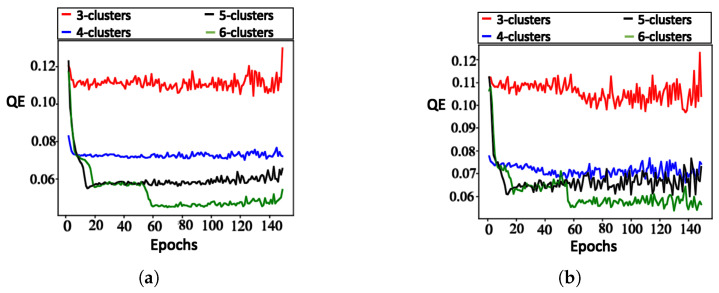
Quantization errors for TA: (**a**) training data, and (**b**) testing data.

**V. Quantization error: **Figure 5 illustrates the training and testing QEs of all 4 SOM configurations. In this figure, each NN shows a significantly low quantization error, with the lowest observed in the training data. The NN with 6 outputs has the smallest error (0.04 in training, 0.053 in testing). The errors were larger for the NNs with 5, 4, and 3 outputs, respectively, with errors increasing as the number of output neurons (clusters) decreased.

The results confirm that QE minimization leads to stronger grouping of the data. This means it produces numerous clusters where the data points are closer to the cluster centroids.

**VI. Discriminant score:**

**Figure 6:**
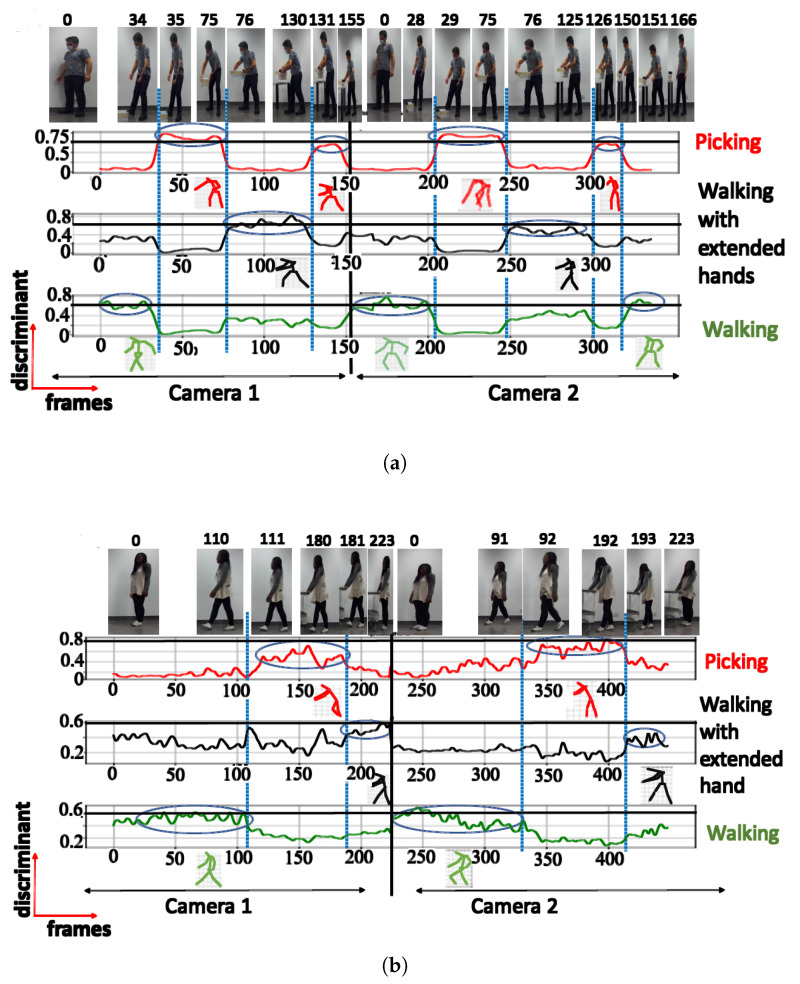
Discriminant score plot of the NN trained for 3 outputs, using the cosine distance: (**a**) TA activity, (**b**) UA activity.

**A. NN trained using cosine distance:** For the architecture with 3 clusters, the following postures were indicated: ‘walking’, ‘picking’, and ‘walking with extended hands’, with a maximum score of 0.97 (see Figure 6a). When testing with a configuration of 3 clusters using the UA activity, the same 3 groups of postures were also recognized (see Figure 6b), as shown in Table 2. The postures represented by the highest peaks in the discriminant score plots exhibit the highest discriminant values and are highlighted with circled peaks for identifying the winning postures in the activity’s particular frame range.

**Figure 7:**
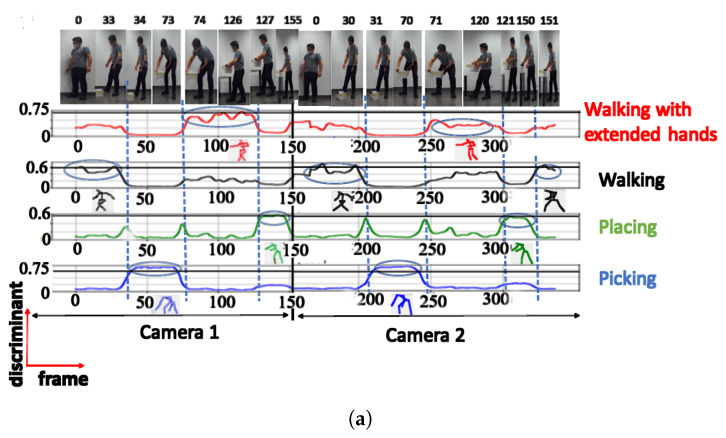

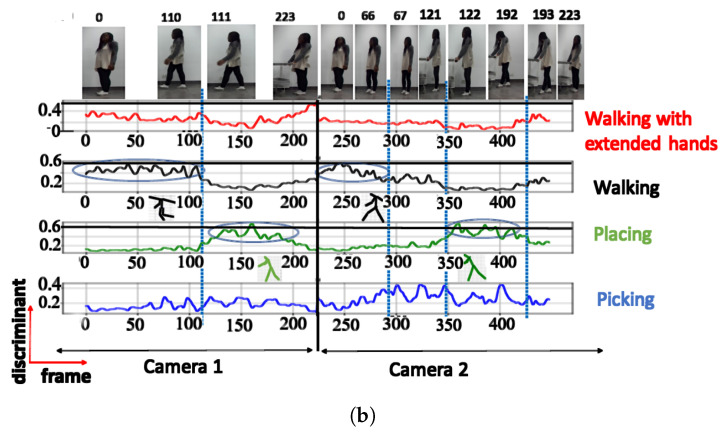
Discriminant score plot of the NN trained for 4 outputs, using the cosine distance: (**a**) TA activity, (**b**) UA activity.

The NN configuration with 4 clusters successfully identified 4 distinct postures during testing, namely ‘walking with extended hands’, ‘walking’, ‘placing’, and ‘picking’. For these postures, the discriminant score was high, reaching 0.9 (Figure 7a).

When testing with UA activity, the NN configuration with 4 clusters only recognized 2 posture classes (‘walking’ and ‘placing’), achieving a maximum discriminant score of 0.68 for ‘placing’ (Figure 7b). The ‘walking with extended hands’ posture was not recognized in this case, despite having a short instant discriminant score of 0.56.

**Figure 8:**
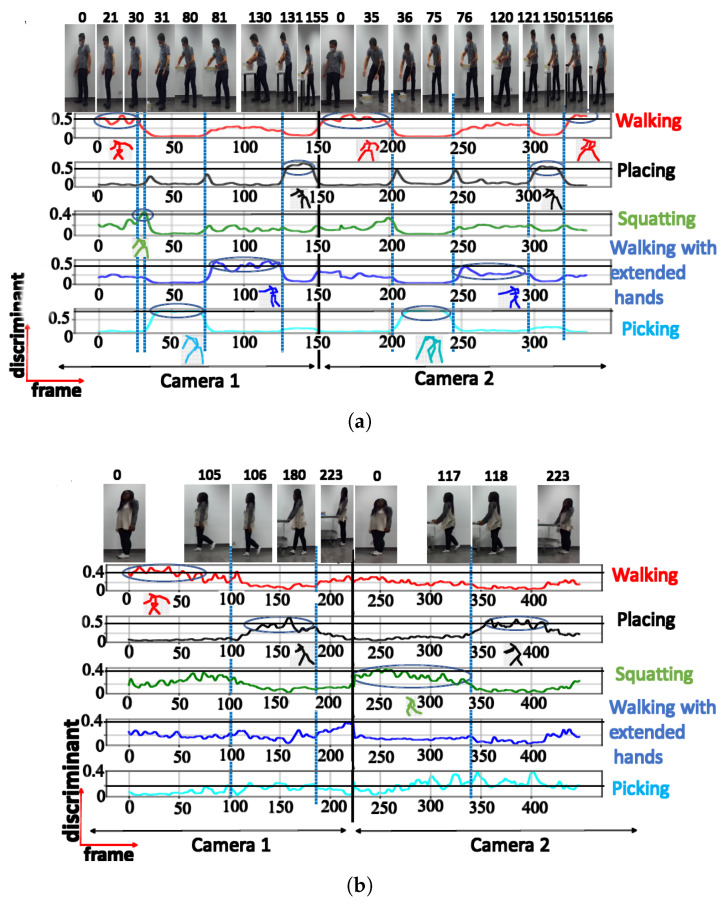
Discriminant score plot of the NN trained for 5 outputs, using the cosine distance: (**a**) TA activity, (**b**) UA activity.

The NN configuration with 5 clusters effectively identified the same postures as the configuration with 4 clusters, but it also included a fifth posture (squatting). However, this additional posture exhibited a low discriminant score of 0.44 and was visible in only a limited number of frames, as illustrated in Figure 8a.

When testing with the UA activity, the NN configuration with 5 clusters recognized 2 distinct posture classes, with the highest class achieving a maximum score of 0.65

**Figure 9:**
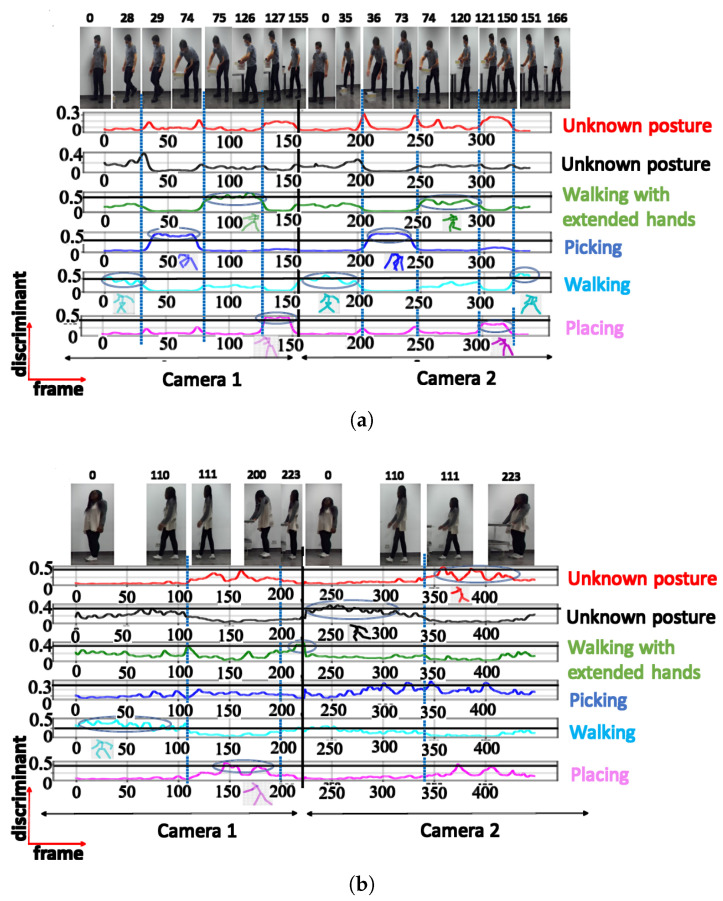
Discriminant score plot of the NN trained for 6 outputs, using the cosine distance: (**a**) TA activity, (**b**) UA activity.

Upon testing the NN configuration with 6 clusters, 4 posture classes (referred to in Figure 9) out of the 6 selected clusters were recognized. This is consistent with the results obtained in the configurations with 4 and 5 clusters. The remaining 2 clusters could not be readily linked to specific postures and were labeled as unknown. The highest discriminant score recorded for the recognized postures was 0.82. Upon testing the UA activity, the neural network successfully recognized 2 postures.

Table 2 displays the highest discriminant scores achieved for the evaluated neural network architectures utilizing cosine distance during training.

**Figure 10:**
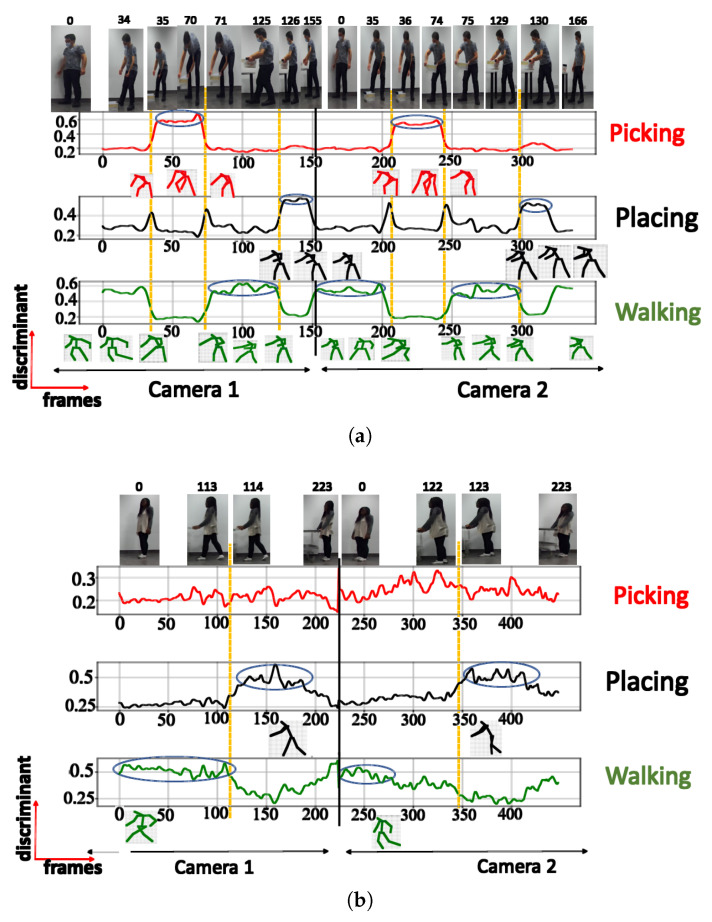
Discriminant score plot of the NN trained for 3 outputs, using the Euclidean distance: (**a**) TA activity, (**b**) UA activity.

**Figure 11:**
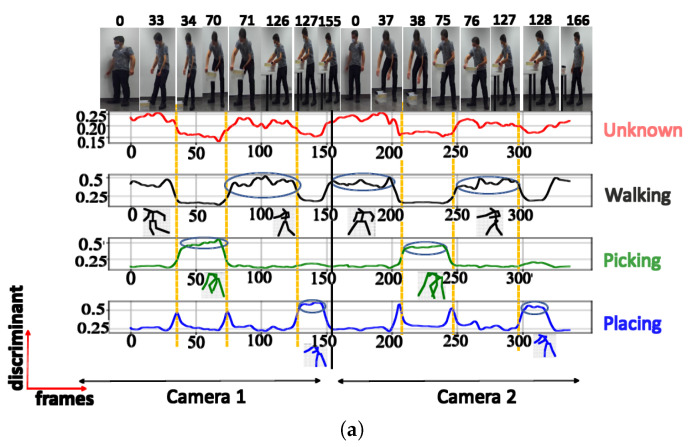

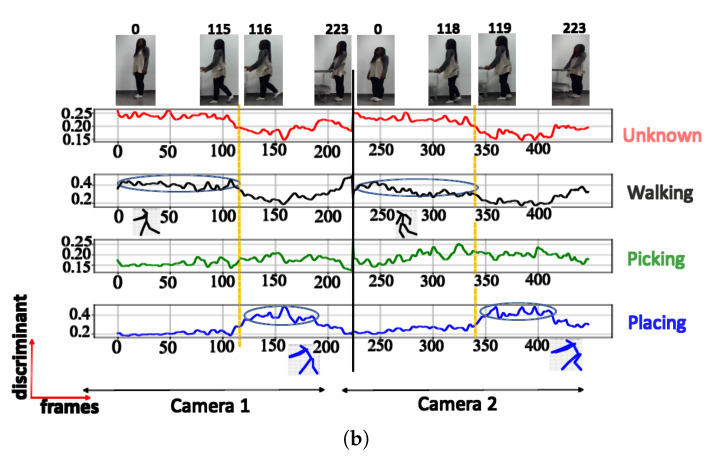
Discriminant score plot of the NN trained for 4 outputs, using the Euclidean distance: (**a**) TA activity, (**b**) UA activity.

**Figure 12:**
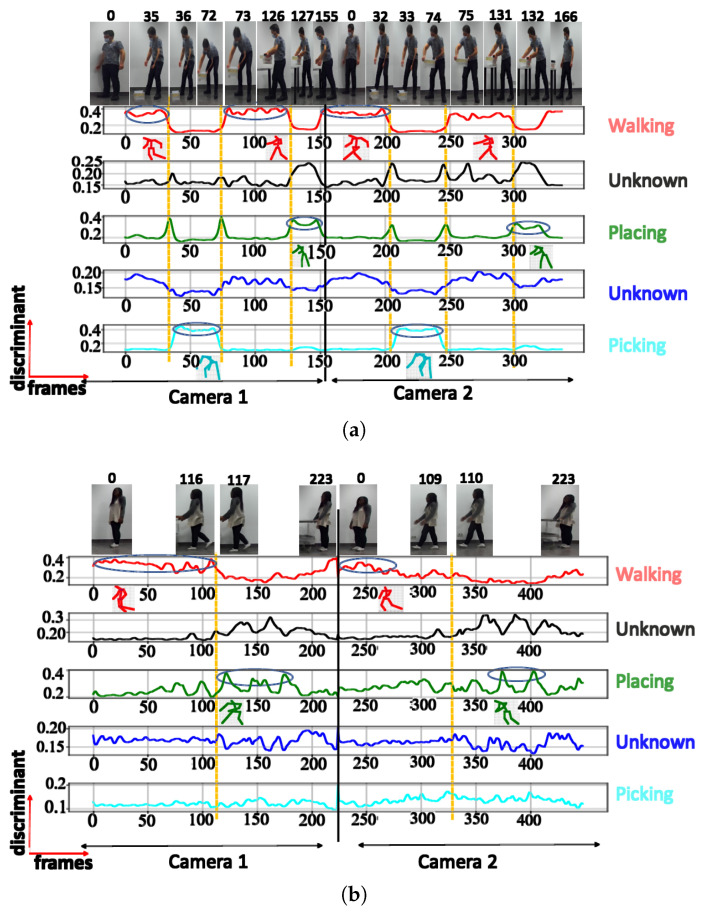
Discriminant score plot of the NN trained for 5 outputs, using the Euclidean distance: (**a**) TA activity, (**b**) UA activity.

**Figure 13:**
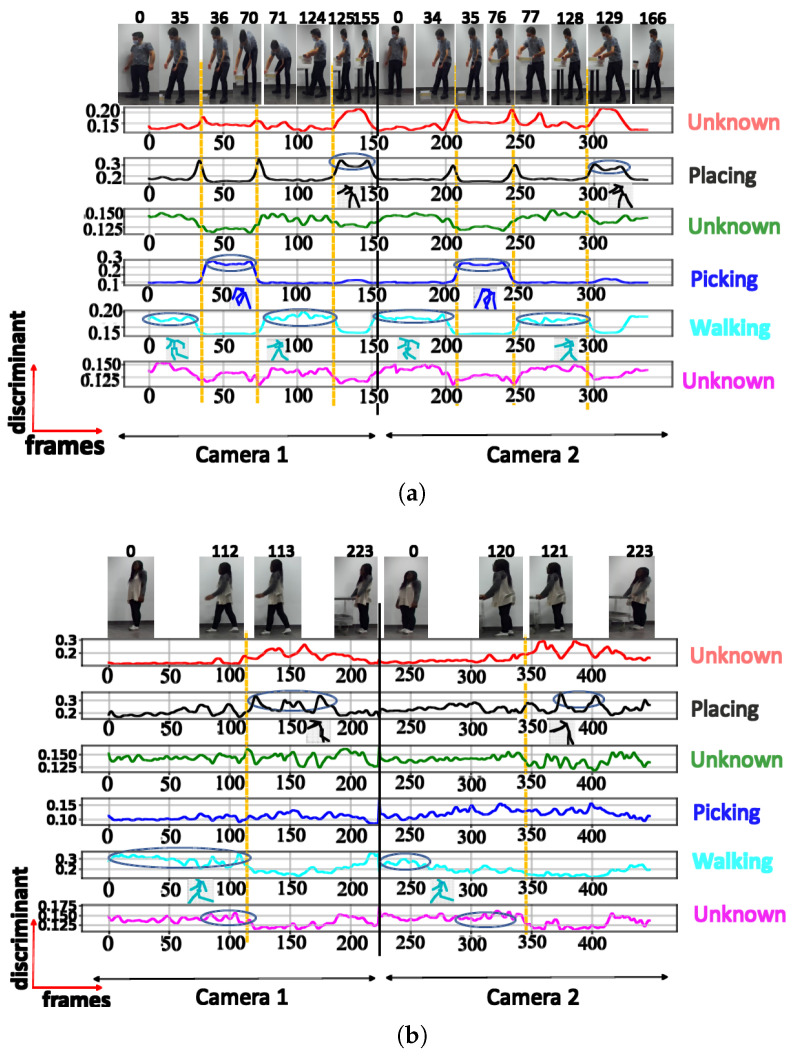
Discriminant score plot of the NN trained for 6 outputs, using the Euclidean distance: (**a**) TA activity, (**b**) UA activity.

**B. NN trained using Euclidean distance:** When the neural network was trained using the Euclidean distance, the discriminant score plots consistently identified 3 posture classes, irrespective of changes in the number of output neurons (or number of clusters). Figure 10, Figure 11, Figure 12 and Figure 13 show the discriminant score plots for 3, 4, 5, and 6 outputs. The neurons that did not win the competition are tagged as ‘unknown’; hence, they do not represent any posture in the activity. It is worth noting that when using an activity not included in the training set (UA), the results are more consistent compared to those from the NN based on the cosine distance metric. For all 4 configurations, the same 2 clusters of postures are identified.

**Figure 14:**
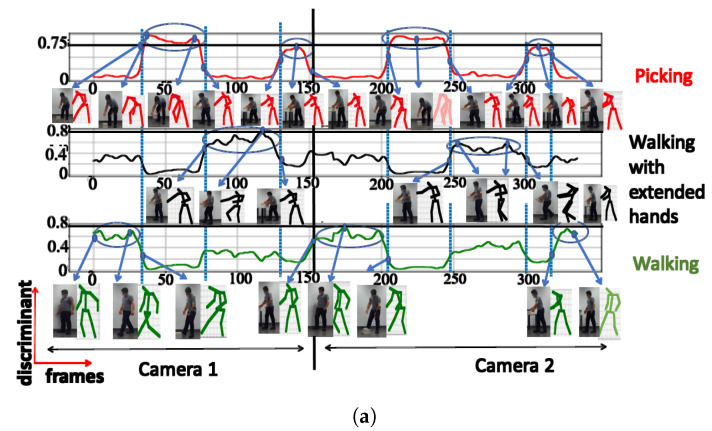

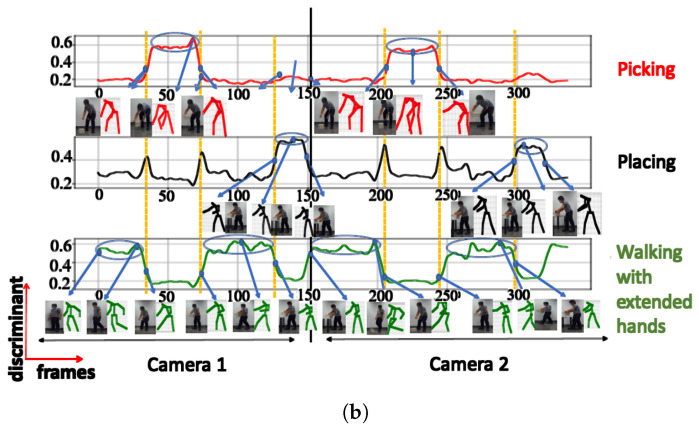
Comparative discriminant score plot for TA clusters: (**a**) cosine, (**b**) Euclidean distances.

**C. Comparison between Euclidean and cosine distances:** Figure 14 illustrates the posture classification with different boundary characteristics and discriminant scores for the NN trained using both the cosine distance and Euclidean distance. Regarding the cosine distance, the postures with semantic meanings ‘walking’ and ‘walking with extended hands’ are both considered to be in the same class, while the 2 other clusters belong to the classes with semantic meanings ‘picking’, and ‘placing’. This suggests that when using the cosine distance for classification, the decision is predominantly influenced by the inclination of the human trunk. During activities like walking or walking with extended hands, the trunk maintains an upright position.

In contrast, during actions like placing and picking, the trunk leans forward to varying degrees. The NN trained using the Euclidean distance, however, groups the postures with the semantic meanings ‘picking’ and ‘placing’ in the same cluster. The remaining clusters belong to the classes with the semantic meaning ‘walking with extended hands’ and ‘walking’. Therefore for the NN trained using the Euclidean distance, the key factors used for clustering consist of the hand and trunk positions. During the ‘picking’ and ‘placing’ actions, individuals lean their trunks forward while extending their hands. In contrast, when walking, they maintain an upright trunk with their hands swinging by their sides. For the ‘walking with extended hands’, even though the trunk remains upright, the hands are extended outward. The arrows at the outermost positions mark the boundary postures between clusters, signaling transitions to the subsequent cluster. The central arrow points to the “key” posture within the cluster.

This results in 3 distinct postural combinations based on the positions of the trunk and hands. These phenomena can be observed in both the stick diagrams and images in Figure 14. The postures at transition points, the maximum score values, and the key class postures have all been illustrated.

In general, the NN exhibited a higher discriminant score when tested with the TA activity than with the UA, which was anticipated. This outcome can be attributed to the NN’s prior exposure to similar samples from the TA during training. Nevertheless, even for the TA activity, the discriminant scores remained notably high, indicating the NN’s proficiency in distinguishing and tracking human postures. The posture transitions between clusters are illustrated by the steep edges marked by vertical lines across the graphs. At this point, there is a rapid decrease in the discriminant score of the current winning cluster and a simultaneous increase in the discriminant score of the next winning cluster.

The NN trained using the cosine distance with 4 outputs surpasses all other configurations due to its high discriminant score, adaptability, and consistent class separation. Thus, if the testing data features are subject to some changes, the network still effectively recognizes the postures. This adaptability results from the self-organizing map network concept, which focuses on the general characteristics of the data structure, and not on very specific data attributes. When choosing the number of clusters based only on the largest values (see Figure 7, Figure 8 and Figure 9), 3 clusters should be used. However, looking at the discriminating score (TA) plots for the 4 clusters, we can see that for all clusters, the temporary peaks of each trajectory have relatively high values, and each distinguished posture (temporary peak) lasts for a significant amount of time. This is not the case for 5 clusters. The score values here are much smaller than in the previous cases, and each DS trajectory is flatter, which means less variation in clustered postures. This phenomenon deepens for 6 clusters. Based on these results and avoiding the omission of key postures, it was decided that 4 clusters should be selected. The above considerations show that when deciding on the appropriate number of clusters, the range of clusters indicated by the expert should be examined, and then, based on the trajectory and the value of the score, the appropriate number of clusters should be selected.

### 6.3. Discussion

The methodology presented for determining the most appropriate number of clusters in human posture classification has demonstrated promising results. It offers a balance between computational efficiency, granularity of postural representation, and distinction, effectively addressing major challenges in the field. However, evaluating clustering quality using CVIs can be influenced by factors such as data distribution [24,31,54], unbalanced cluster representation, and diverse recommendations from different CVIs. They sometimes suggest different optimal cluster numbers, which adds to the complexity of determining the best decision based on the data distribution. An arbitrary decision on the number of clusters, which does not take into account the specificity of the problem, may result in losing information about data features in the clustering process [55,56]. For general types of datasets, mitigating the data distribution challenges often requires specific strategies. One strategy for solving the problem is to more densely sample those fragments of data that show greater dispersion [57,58]. Another approach is to use multiple clustering processes with different cluster numbers [59,60]. However, it is worth noting that the presented work is rather problem-oriented when compared to universal data processing research. According to existing literature, the decision on the appropriate number of clusters should be made based on the unique requirements and specifics of the problem [61,62,63]. The most important involves the use of domain knowledge, i.e., taking into account the clustering aim. This means that dedicated approaches should be preferred by avoiding decision-making based solely on general-purpose scores. For example, understanding that some types of postures are inherently rarer can help in making informed clustering decisions. This is an important aspect of the proposed discriminant score as it allows detecting the relevant postures, which are represented by few data but are indicated by higher score values.

The results obtained using various scores, namely the discrimination score, silhouette coefficient, Dunn index, Davies–Bouldin index, and Calinski–Harabasz index, together with quantization error, show that each score focuses on different aspects of data clustering.

The silhouette coefficient evaluates the degree of similarity of an object to the centroid of its own cluster in relation to its similarity to centroids of other clusters. Thus, when an analyzed sequence contains postures that change in broad ranges, this coefficient will tend to show fewer clusters than other scores. This can make it difficult to distinguish between activities characterized by quite similar postures because only postures with big differences will be considered as being different. The Davies–Bouldin index has the opposite feature. It is based on the idea that good clusters are those that have low within-cluster variation and high between-cluster separation. In this case, there is a risk that virtually identical postures, albeit realized by different individuals or in different conditions, will be assigned to different clusters (due to higher variation in the data); this is not appropriate. It means that this index is sensitive to noises and outliers in the data. Unfortunately, when recording human activities in real-life conditions, disturbances are not avoidable.

The Dunn index is the ratio of the minimum distance between clusters to the maximum cluster diameter. This indicator is higher for clearly ‘discretized’ sets of postures. Since changes in posture are continuous, such cases are not feasible, can occur for temporarily obstructed views, etc. This index is denoted as the worst-case indicator. Therefore, when considering the task of classifying the postures, the Dunn index should be approached with reserve. The quantization error should be considered as the training process evaluator rather than the clustering quality score that is applicable to the trained NN. Additionally, it favors a strong concentration of data around cluster centroids, which is feasible for a larger number of clusters. Therefore, its utility in deciding the expected number of postures is limited. It shows a similar weakness to the Davies–Bouldin index when considering posture classification.

The Calinski–Harabasz index identifies several clusters that are both compact and sufficiently distant from each other. It is also known as the variance ratio criterion, and it measures how similar an object is to its own cluster compared to other clusters. Considering the posture recognition task, where the goal is to group significantly similar postures with reasonable (not excessive) separation between groups, this aim is reasonable. The logic behind this index is similar to the logic used in establishing the discriminant score; however, the index provides only a single value.

The discriminant score provides a value for each data point (each posture). This is the normalized inverse of the posture distance from the centroid of the cluster. This value is divided by the sum of the minimum distances between this data point and the centroids of all clusters. The score shows how similar the posture is to the postures that make the cluster centroids, taking into account the distribution of clusters (this is included in the denominator). Unlike other scores, the discriminant score allows following the dynamics of the classification, as it shows how the similarity of postures to the postures constituting the cluster centroids change over time. Thus, the score facilitates a fully informed decision regarding the number of clusters that ensure appropriate differentiation of postures, as indicated by higher values of the score. Choosing the right number of clusters is not reliant on a single value, unlike other scores that overlook the finer details of the data structure.

In the given example, the silhouette coefficient suggested 3 clusters, while the Dunn index and the Davies–Bouldin index favored 5 clusters. The quantization error suggested 6 classes. The discrepancy in these results highlights the challenge of determining the ‘optimal’ number of clusters.

The decision to choose 4 as the most suitable number of clusters was based on the discriminant score and the Calinski–Harabasz index. Both scores take into account the balance between clear class separation, data adaptability, and consistent representation of data features. Furthermore, the discriminant score is particularly relevant because of its ability to detect posture similarities, express them numerically, and show the transformations in the form of trajectories.

It must be emphasized that, in the analyzed example, the discriminant score was relatively high regardless of the number of clusters; this indicated that essential data features were learned, and generalization was performed well. Since the DS assigned a value to each posture, based on closeness to the respective cluster centers, choosing the right number of clusters (4 in this case) was not based on a single value, as was the case with other scores that lost data structure details. Unlike other indicators, the discriminant score enables the tracking of classification dynamics. It illustrates how the similarity of a posture to cluster centroids evolves over time. This allows for a well-informed decision regarding the optimal number of clusters that ensures appropriate differentiation of postures (through higher values of the indicator). Hence, selecting the most appropriate number of clusters based on this score offers resistance to disturbances or variations in posture during activity performance. This is because the selected number of clusters ensures appropriate separation. The discriminant score also expresses the generalizability of the SOM, as it is able to recognize postures taken in previously unseen activities (e.g., the UA activity). Therefore, a well-chosen number of clusters (with potentially high discriminant scores) enables the recognition of the same postures (as well as similar postures) in activities that were not previously considered.

## 7. Conclusions

Utilizing NN with unsupervised self-organizing mapping to cluster data eliminates the need for arbitrary and time-consuming labeling of training data. However, the choice of the number of clusters is crucial here. Our previous study [47] explored the challenges of manual data labeling by human experts for clustering, as the range of activities expanded. This further indicated the necessity for the methodology presented in this work, which aligns with earlier expert opinions and allows accommodating a broader array of human postures and cultural considerations. The presented research focuses on the utility of a set of scores for determining the appropriate number of clusters for the human posture classification task. It is rather problem-oriented compared to data-oriented research. Commonly used scores are broadly applicable and are not adjusted to the particularity of data. Relying solely on general scores is definitely not sufficient. For instance, using the Dunn index or the Davies–Bouldin index can reduce the granularity of posture differentiation due to data distribution sensitivities [24,31,54,55,56]. The proposed discriminant score provides a dynamic perspective on posture classification over time, emphasizing the importance of selecting an adequate number of clusters that preserve the data structure details. This is in contrast to other scores that yield a singular value. The unsupervised self-organizing map is effective at recognizing postures across diverse activities, offering the necessary adaptability for real-world applications.

For practical reasons and to avoid excessive calculations, only examples using small subsets of data are discussed in this article. This limitation does not reduce the substantive significance of the work and the validity of the presented analyses. The specificity of the clustering application, namely the classification of human postures, is of primary importance. The objective of developing a methodology to select the appropriate number of clusters for grouping and, thus, identifying postures has been achieved. This methodology is not sensitive to biases, etc. Therefore, even with a limited dataset, the results are of significant importance for the posture classification task.

The contribution to the state of the art can be summarized as follows:

- The introduction of a new clustering quality assessment namely, termed the discriminant score.
- Comparative studies of clustering scores, considering the posture classification task, with an indication of the advantages and disadvantages of each of them.

The autonomous recognition of human postures is necessary across many domains. For example, in healthcare [64,65], it is used for monitoring changes in a patient’s posture and providing automated assistance for correction. In elderly care [66,67], changes in posture may indicate discomfort or health problems. In robot-assisted human activities [68,69,70], it is essential for understanding what a person is doing based on their sequence of postures, offering assistance when necessary.

Our research is focused on learning by observing human actions [18,19]. In our future work, we plan to expand our datasets and train the neural networks, taking into account multiple activities with many postures. The most appropriate number of clusters will be chosen using the discriminant score. After training and testing using big data, we aim to establish an autonomous system capable of recognizing activities from a sequence of postures. The ultimate goal is to create an ‘intelligence’ for robots that will not only be able to identify human postures but also recognize and anticipate human actions.

## Figures and Tables

**Table 1:** Basic notation.

Notation	Description
*N*	total number of samples
*M*	total number of clusters
$f_{j}$	*j*-th data point,
	*j* = 1,…, N
$c_{m}$	*m*-th cluster,
	*m* = 1,…, M
$K_{m}$	number of data points
	assigned to cluster $c_{m}$
$f_{k}^{c_{m}}$	data point assigned to $c_{m}$,
	$k=1,\ldots ,K_{m}$
${\mathrm{cc}}_{m}$	centroid of cluster $c_{m}$
cc	centroid of all clusters
$w_{m}$	the weights vector of the *m*-th
	output neuron
$d^{Me}\left(x,y\right)$	the distance between *x* and *y*
	obtained with Me={Cos,Euc}
	metrics, $x=\{f_{j},c_{m}\}$
	$y=\{w_{m},c_{n}\}$
$d_{min}^{Me}\left(x,y\right)$	minimum distance between x and y
|•|	absolute value of •
||•||	Euclidean distance

**Table 2:** Maximum discriminant scores for the TA and UA activities.

Activity	Number of Clusters	Semantic Meaning	Max DS
Training activity (TA)	3	Walking	0.81
		Picking	0.97
		Walking with extended hands	0.78
	4	Walking	0.71
		Picking	0.90
		Walking with extended hands	0.78
		Placing	0.60
	5	Walking	0.61
		Squatting	0.44
		Picking	0.84
		Walking with extended hands	0.63
		Placing	0.64
	6	Walking	0.66
		Picking	0.82
		Walking with extended hands	0.70
		Placing	0.59
		Unknown posture	0.32
		Unknown posture	0.39
Non-training activity (UA)	3	Walking	0.60
		Picking	0.84
		Walking with extended hands	0.65
	4	Walking	0.59
		Picking	0.44
		Walking with extended hands	0.56
		Placing	0.68
	5	Walking	0.54
		Squatting	0.43
		Picking	0.38
		Walking with extended hands	0.42
		Placing	0.65
	6	Walking	0.48
		Picking	0.32
		Walking with extended hands	0.41
		Placing	0.56
		Unknown posture	0.53
		Unknown posture	0.42

## Data Availability

Source data are available upon request, subject to approval by the laboratory.
